# A Diagnostic Method Based on Active Thermography for the Degradation Assessment of Power Plant Boiler Tubes

**DOI:** 10.3390/s22218401

**Published:** 2022-11-01

**Authors:** Sławomir Zator, Michał Tomaszewski, Mirosław Lasar

**Affiliations:** 1 Faculty of Production Engineering and Logistics, Opole University of Technology, Ozimska 75, 45-370 Opole, Poland; 2 Department of Computer Science, Opole University of Technology, Prószkowska 76, 45-758 Opole, Poland

**Keywords:** active thermography, noninvasive diagnostic method, power plant boiler, furnace shield, excessive degradation

## Abstract

Proper maintenance management of power infrastructure requires inspections, in order to gather knowledge about the facility’s current condition. For this purpose, periodic diagnostic tests are carried out, not only to determine the current state, but to also predict future conditions, and subsequently plan for the scope of necessary repair work. Currently, in the case of heat screens of power boilers, the diagnostic process takes many days, is very expensive, and usually does not cover the entire screen area. Therefore, it is necessary to develop new, noninvasive diagnostic methods. This study presents the concept and research for an alternative method of locating places with excessive energy boiler screen degradation that require replacement. It was assumed that the new method should be fast, require no scaffolding assembly, and permit checking of the entire screen surface; this is unlike the methods used currently, which require manual checking of selected screen tubes. The proposed method is based on active thermography, in which heat flux is forced by the liquid flowing inside the screen. Tests were carried out based on a model of an axial-symmetric system in the form of a tube, with controlled reductions in the wall thickness. An experiment was carried out many times by recording the pipe surface temperature in many characteristic places (different thicknesses of the tube walls) with a thermal imaging camera. A temperature change was forced by a controlled flow of hot or cold water. The methods of analysis were proposed and verified, allowing firstly, detection of places with a reduced wall thickness, and secondly, estimations of the wall thickness (i.e., excessive degradation). For the best-proposed model (one of the four analyzed), all of the thickness changes were detected, and the limit error of thickness obtained was 0.3 mm.

## 1. Introduction

Managing a modern technical system is a very complex process. It requires, among other things, activities such as the following:

- periodic inspections—allowing for the assessment of the actual condition;
- scheduled maintenance—keeping the device in full production readiness;
- repairs—restoring equipment to a condition that allows it to compete with other companies’ systems.

**Figure 1:**
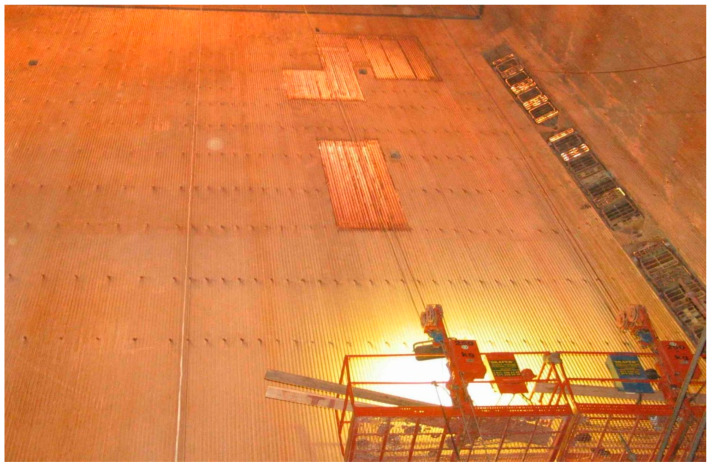
Boiler interior, showing scaffolding and the replaced screen fragments.

The evaporator is the central part of the boiler, inside which pulverized coal is burned, and where the temperature is the highest. Water turns into vapor in the tubes of the evaporator walls. The walls of the evaporator are called screens, and they are exposed to the most significant degradation that is associated with low oxygen corrosion and micro-grinding by coal particles and ash. Therefore, the main diagnostic and repair efforts are directed to this boiler area. The energy screens of a power plant’s boiler undergo regular periodic diagnostic inspections, in order to observe the combustion process’s effects. Diagnosing the tubular boiler elements is aimed at preventing failures that may disrupt or completely stop the operation of the power plant block. The photo in Figure 1 shows the inside of a power plant’s boiler during periodic, planned renovation.

Boiler screens, which are made of a series of pipes (224 for the rear screen and 120 each for the side screens), are subject to solid destructive factors, and the occurring corrosion phenomenon significantly reduces the durability of the pipes; their corrosion may lead to failure of the facility. The tubes of new screens are 5 mm thick.

Currently, the diagnostics of thermal screens is preceded by a thorough, mechanical cleaning of their surfaces. After the tubes are cleaned, the so-called grinding for measuring purposes is performed. For economic reasons, the measurement values are interpolated during the renovation; not every pipe is measured, only every fifth or tenth pipe, at vertical intervals of 2–3 m. Based on the obtained wall thickness distribution, the screens are replaced in areas where the wall thinning is less than 3 mm. This process is very time-consuming, as the boiler screen, e.g., BP-1150, is almost 90 m high.

**Figure 2:**
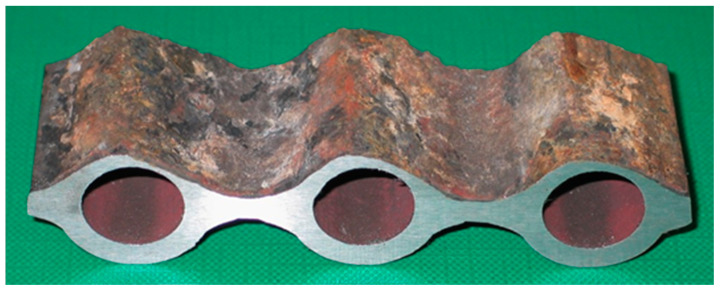
Cross section of a screen fragment that was damaged by coal and ash particles as a result of corrosion and fire degradation.

During diagnostics, measurement data are collected in a spreadsheet. The photo in Figure 2 shows the cross section of a screen fragment.

Correct maintenance management requires knowledge about the facility’s current condition. In the case of thermal boiler screens, the diagnostic process takes many days to complete the assessment, and does not cover its entire surface. Therefore, it is necessary to develop new, noninvasive diagnostic (NID) methods that are based on currently available measurement methods, which would significantly reduce the time, and and enhance the quality of diagnostics.

The research presented in this study relates to developing a new method of diagnostics for the OP-650 (BP-1150) boiler evaporator screens.

Mainly before and sometimes after replacing the degraded screen fragments, a tightness test is performed. Water is pumped into the pipes in the entire block of the boiler. Initially, the pressure is low, but increases in the subsequent stage. In the first stage of the process, the water temperature is around 20 °C. In subsequent tests, it is raised by an average of 1–2 °C per hour. During these tests, the patency of all pipes is checked with a thermal imaging camera. Pipes that are clogged have a lower temperature. A similar mode of thermal forcing is proposed for the new diagnostic method. A single tube was used instead of a screen for model tests, in which the same thermal and flow phenomena occur. The model ignores the additional heat flux caused by the transfer between the pipes.

With the conventional approach [1], during the scheduled, periodic shutdown of the power unit, measurements of the thickness of the pipes that make up the walls of the screen are performed on several levels to determine the scope of their replacement, in order to avoid failures that may result in unplanned interruptions and financial losses. This results in a total of about 6.5 thousand measurement points for the entire screen. On this basis, the renovation area is designated and replaced [2]. In this case, these will be all the pipes with a less-than-acceptable thickness, and those that will be replaced for other technical reasons.

The study in the second section describes methods that are, or can potentially be used for diagnostics, including thickness measurements of homogeneous materials, mainly metals. The first section describes the test stand, its equipment, and software functions. Examples of the measurement results are shown. The fourth section presents the mathematical model of the temperature distribution in a single tube, caused by pulse temperature stimulation. The fifth part presents the adopted models and the obtained results, which enable the forecasting of changes in the wall thickness of the pipes. The last section is a summary, and the application possibilities of the developed method.

## 2. Applied Diagnostic Methods

### 2.1. Ultrasonic Methods

A review of non-destructive diagnostic methods for fireside corrosion risk assessment of industrial boilers is presented in article [3]. Many potential methods are listed and described, but only a few have potential for practical use. Moreover, review article [4] presents pipeline inspection methods that can be used in boiler diagnostics. Measurements of *t* thickness of the wall tubes are carried out during boiler outages, since surface cleaning is usually necessary before the inspection.

A method that is commonly used is the classic ultrasound method. This ultrasonographic method utilizes the differences in the velocity of mechanical vibration movement in the materials studied, and the reflection of waves at media boundaries. In ultrasonographic measurement, an ultrasonic impulse is sent, which bounces off the sample edge, and returns to the receptive head. Knowing the speed at which the wave is moving in a given medium, and measuring the times of movement of the reflected wave, it is possible to estimate the thickness of the measured sample, and to locate defects in its structure [5]. One standard procedure for measuring the tube thickness is to grind the fireside of the tube, and use an ultrasonogram to determine the wall thickness. This approach provides good results; however, due to the laborious preparation process (sandblasting, scaffolding setting), the measurement process takes a long time, and is costly. Moreover, the conventional ultrasonic method requires a liquid couplant, which is difficult to automate [6].

The EMAT (electromagnetic acoustic transducer method) is the second well-known method. The EMAT transducer has a strong permanent magnet, usually of the neodymium type, and a spiral coil supplied with a high-frequency current, which induces eddy currents. Two mechanisms between an applied magnetic field and induced eddy currents produce an elastic wave: the Lorentz force and magnetostriction. The Lorentz force mechanism works well on boiler tubes, but requires much higher magnetization and systems, which is why they are heavier and more costly than their magnetostrictive counterparts. Since the steel surface has low magnetostrictive properties, clean tubes cannot generate sufficient EMAT signals. However, if the surface (tightly bonded scale) has a significant magnetostriction coefficient [7], the EMAT transducers can work with general-purpose ultrasonic instruments, with certain modifications. The measurements can be carried out quickly, without the need of a liquid couplant (as with the ultrasonic method). Compared to the classic ultrasonic method, the EMAT method allows for contactless measurement, and has less sensitivity to the state of the surface. Some limitations [8] of this method are the influence of the head distance, and the ash deposits’ chemical composition and structure, on measurement accuracy.

### 2.2. Magnetic and Electromagnetic Methods

Potentially two of many electromagnetic measurement methods employed in the study of materials utilized in the construction of industrial-scale boilers can be used: the magnetic flux leakage (MFL) method, and the saturated low-frequency eddy current (SLOFEC) method.

The MFL method employs the magnetization of the material studied to the saturation level, as well as the measurement of the magnetic field corona [9]. Although this detection technique has been known for a long time, research is ongoing on it to determine the structure of defects. In order for the test to be carried out properly, calibration tests are necessary, whose task is to adjust the measurement system to the specifics of the tested object. The measurement accuracy constitutes the limitation of the method. Material losses are identified without information on which side of the sample the failure is located.

The SLOFEC method uses eddy currents and a constant magnetic field [10], in contrast to the classic method that employs eddy currents, which is unsuitable for studying ferromagnetic materials. The constant magnetic field improves the eddy current penetration depth. The analyses of the eddy current field, especially in amplitude and the signal phase, allow for assessment of tube wall thickness loss. Unfortunately, the SLOFEC method is not an absolute wall thickness measurement technique. Calibration with a comparison technique is necessary.

Another non-contact inspection method is the pulsed eddy current technique (PECT). It is a relatively new technique used for the inspection of ferrous materials. It provides much more information when compared to conventional eddy current techniques, due to broadband analysis [11]. The PECT can be applied to measuring wall-thinning pipe walls when its properties are well-known.

### 2.3. Ionizing Radiation Methods

Utilizing ionizing radiation is a potential method for tube thickness analysis. However, it has a significant limitation, as the tube must be located between the source and receiver of the radiation. In practice, this requires the synchronized movement of heads on both sides of the analyzed tube. In the case of screens with insulation, this may cause some difficulties, especially when additional angular exposure is required. They detect corrosion and erosion defects on both sides of the outer and inner tube surface [12]. This method would allow for the measurement process to be automated. It would also have minimal costs associated with the preparation of the surfaces for measurement. Using dual-energy radiation and neural networks to measure scale layer thickness in oil pipelines was shown in article [13]. Determining the exact value of the diameter inside the pipe was very important to minimize the energy required for pumping.

### 2.4. Infrared Radiation-Based Methods

Temperature is one of the most common indicators of the structural health of equipment and components [14]. Often, damaged components and defective parts of machines contribute to temperatures being different from regular operation. Currently, infrared thermography (IRT) is an excellent tool for monitoring the condition of an object where the temperature distribution not only identifies, but even predicts the source of a failure [15].

Thermographic methods are divided into those that are passive and active. In passive methods, only the object’s surface is recorded and analyzed. In active thermography, the tested material is heated by a forced thermal impulse, and the surface temperature is recorded using a thermal imaging camera. Active infrared thermography is a fast-developing non-destructive technique that uses different external excitation sources. In the beginning, long-wave radiation sources were used, such as halogen lamps, then lasers. The newest variants use electromagnetic stimulation: induced eddy currents or microwaves [16]. In order to generate heat in the component under inspection, ultrasonic waves are also used. The heat dissipation rate through areas of the material of different thicknesses varies, depending on the condition of the observed object.

Due to the duration of the excitation and its shape, active thermography is divided into short pulse, long pulse, and phase thermography, with a sinusoidal shape, also known as the lock-in method. Lock-in thermography was used for segmenting impact damage of carbon fiber-reinforced plastics, in fields such as aerospace [17]. Some studies compared the use of various forcings, trying to identify areas of use. For example, a study [18] showed that the eddy current-pulsed technique was more suitable for metallic material. In contrast, the long-pulse technique was more appropriate for detecting damage in materials with low thermal diffusivity, such as laminates. Recent studies used long-pulse thermography to measure coating thickness unevenness [19]. Step heating thermography is one of the newest versions of long-pulse heat stimulation. This allows for a more accurate determination of small and deep defects, for which a blurring of shape is caused by heat diffusion [20].

Based on the dynamics of temperature changes, it is possible to assess changes in the thickness of boiler screen pipes, and other defects that affect the way heat is conducted in a material. One of the advantages of this method is the ease of automation of the measurement process, and the possibility of obtaining an approximate thickness map of large-area screens after the scanning process is completed [21]. Calibration related to the physical properties of the material under investigation is usually required [22,23].

## 3. Description of the Test Stand

**Figure 3:**
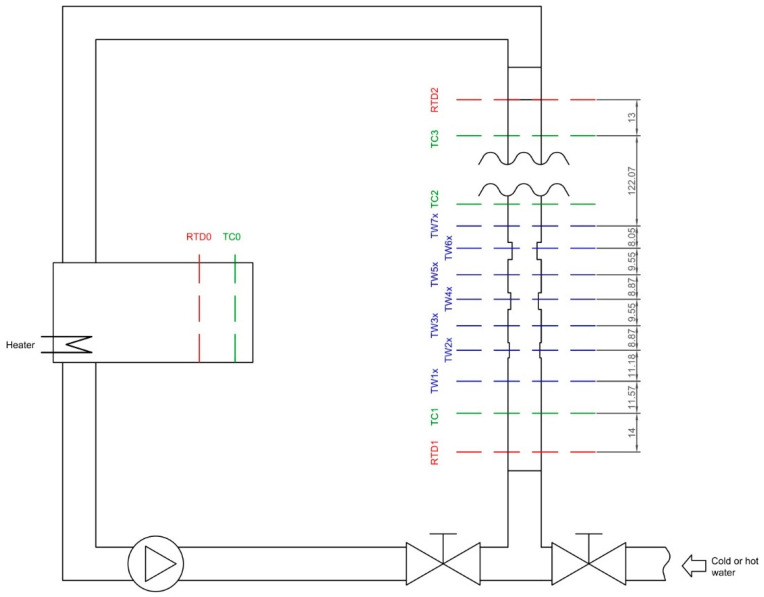
Diagram of the measuring stand, with marked locations of temperature sensors and places of narrowing in pipe wall thickness.

**Figure 4:**
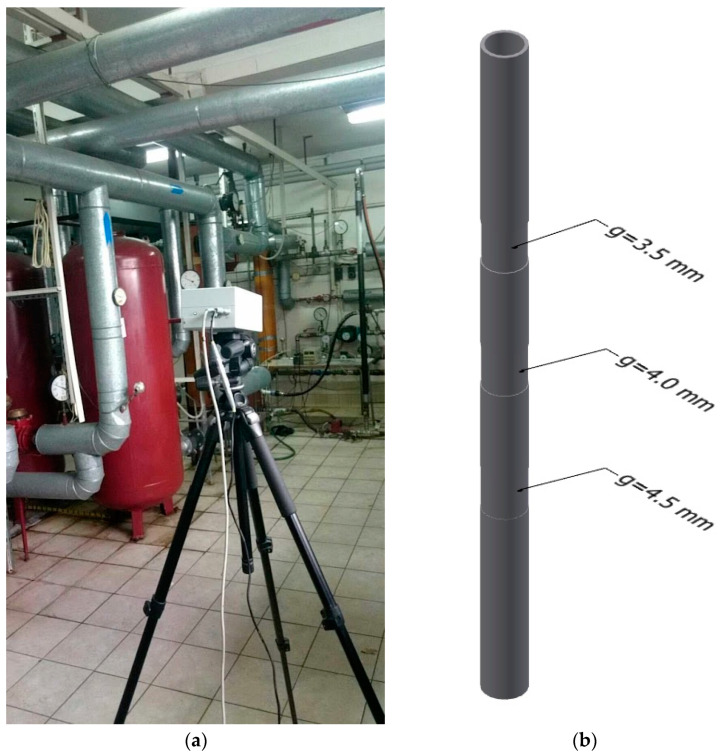
(**a**) Photo of the measuring stand using active thermography, (**b**) vertical pipe with a wall thickness of *g* = 5 mm, with narrowed regions marked.

A measuring stand was built for the research needs; its schematic diagram is shown in Figure 3. The diagram also shows the individual sensor distances used in the mathematical model. The basic version of the stand consisted of a tank with an installed heater with a thermostatic temperature control system, a circulating pump, and the tested object—the measuring section. Water from the measurement section returned to the tank. The valve system also allowed direct water to the measurement section of water from the hot water buffer from the building’s heat node, or cold water from the water supply network. These solutions made it possible to maintain the water at the same temperature over a long period, and to cool the research facility. The test object was a DN50 steel pipe, 2 m long; it was processed on an eccentric lathe (Figure 4b). There were three cuts with depths of 0.5 mm, 1 mm, and 1.5 mm. The thickness g of the ground areas was verified with an Olympus DL 37 Plus ultrasonic thickness gauge. It is a device that is mainly applied to wall thickness measurements of pipes, tanks, pressure equipment, hulls, and other structures (also corroded on the surface).

The device uses single or double heads, which allows for the standard measurement of metal thickness to a resolution of 0.01 mm. A powder coating with a high emissivity coefficient, close to 1, was applied to the entire surface of the pipe.

At the beginning and the end of the facility, Pt100 temperature sensors were installed (labelled RTD1 and RTD2), as well as thermocouples: TC1 (at the beginning), TC2 (in the middle), and TC3 (at the end). The thermocouple TC0 and the resistance temperature sensor RTD0 were installed in the storage tank. The TC0 sensor was used in the heating/cooling controller to stabilize the water temperature in the tank. RTD temperatures were not used in the analyses; they will be used in further research, because sensors of this type are usually installed on real objects. The Lauda UB30JL ultrathermostat was used to calibrate the temperature sensors. The exact locations of the sensors are given in Figure 3. LabVIEW was used to record the measurements. From the hardware side, the NI cRIO-9024 control and measurement set, with the NI 9217, NI 9213, and NI 9481 control modules, was used for data acquisition. The hot ends of the thermocouples were placed on the axis of the tube using a Refco technical endoscope. Pt100 sensors were housed in metal sheaths that were typical for industrial mounting. The application created in the LabView environment controlled the pump, registered the measurements from temperature sensors and the flowmeter, and synchronized the thermal camera VarioCAM. Collected sets of data were saved for further calculations in text files.

The research used the VarioCAM Head (Figure 4a) thermal imaging camera with the following parameters:

- microbolometer matrix of 320 × 240 pixels;
- temperature measurement range from −40 to 1200 °C;
- spectral range from 7.5 to 14 µm;
- temperature resolution of 0.08 K;
- measurement uncertainty of 2 K.

**Figure 5:**
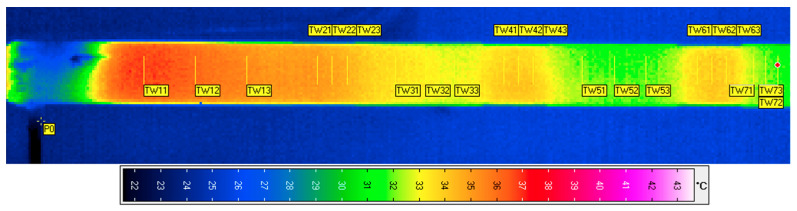
Single thermogram with marked measurement areas.

Figure 4b and the thermogram in Figure 5 indicate areas of reduced thickness. There are places marked as follows:

- thickness reduction by 0.5 mm—TW21, TW22, TW23;
- thickness reduction by 1 mm—TW41, TW42, TW43;
- thickness reduction by 1.5 mm—TW61, TW62, TW63.

The remaining area was at standard pipe wall thickness.

The analysis areas (lines on the thermogram in Figure 5) were 13 pixels, each time on the thermal image. Temperature averaging in these areas partially helped to reduce noise.

**Figure 6:**
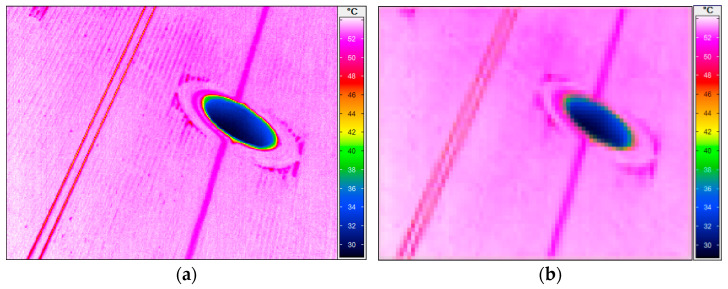
(**a**) Boiler screen thermogram using a telephoto lens 6 × 8, and (**b**) using a standard lens 50 × 68.

Figure 6 shows an exemplary thermogram that was obtained during the tests. A telephoto lens with the following parameters was also used: focal length f = 100 mm, angle of view 8° × 6°). This solution allowed us to increase the resolution in the measured area, since lenses with a standard focal length did not allow for a sufficient resolution of infrared images to identify individual screen tubes.

The configuration of the measuring station allowed us to obtain parameters that confirmed the effectiveness of the proposed method. Based on the registered thermogram sequences, using Irbis, and the VarioCAM dedicated software, timelines of temperature changes were generated for all linear markers, as shown in Figure 5. The results of processing data from thermograms were saved in text files.

**Figure 7:**
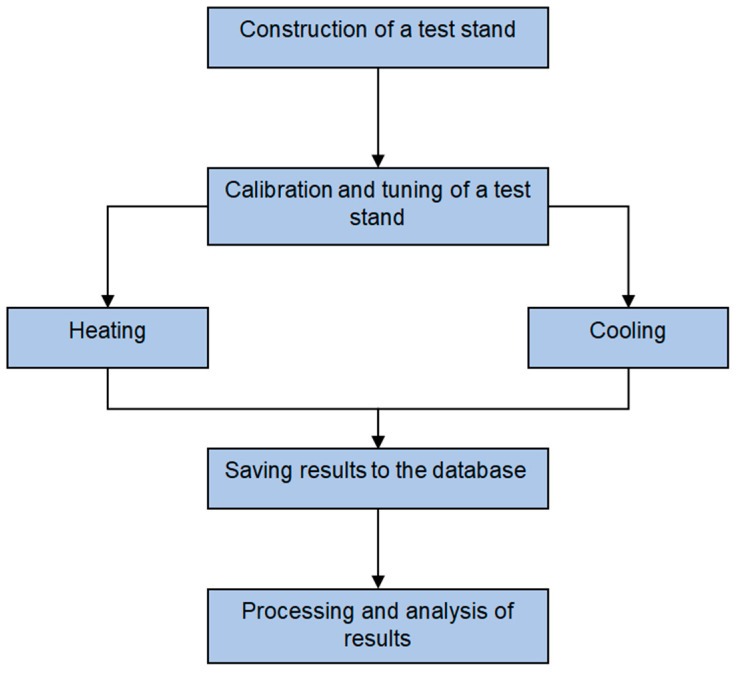
Scheme for implementation of the measurement experiment.

Figure 7 shows the successive experimental stages. After building the stand and calibration, which consisted of temperature sensors and the object’s emissivity, the wall thicknesses of pipes were checked in places of standard thickness, and in places where the cuts were made. Then, the measurements from RTD sensors and thermocouples were interpolated, in order to obtain the same number of samples per time unit as for the thermovision measurements. The acquisition period of temperature and flow was 0.5 s, while those of the thermal images were 0.1 s. Nearest neighbours interpolation was also used during the interpolation of thermograms, in order to obtain their higher resolution [24]. This was the most time-consuming stage of data preparation for further analysis.

The pipe was heated or cooled during the experiments by the water flowing in it. In this way, thirteen measurement series were registered, ten of which were selected for further analysis. The rejected series were too long, or there was a disruption in their registration.

**Figure 8:**
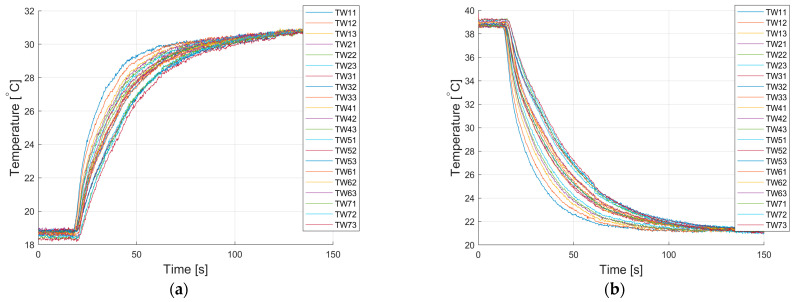
Visualization of measurement results—step responses determined based on measurements with a thermovision camera at the observed measurement points: (**a**) object heating process, (**b**) object cooling process.

Figure 8 shows the responses to the quasi-step excitation that were determined on the basis of the registered series of thermograms during the experiment, for the process of heating and cooling the tested object.

The problem of surface emissivity influence is well-known in all thermographic measurements. However, active thermography may be partially robust to this problem in our case. The determined time constants for selected measuring areas on thermograms are important. They are based on measurements, but even if the surface emissivity coefficient does not correspond to the actual value, this value was maintained throughout the entire measurement; thus, it did not affect the obtained estimation of the model parameters. However, attention was paid to selecting the measurement area in the thermograms. These are lines that are perpendicular to the pipe axis, but not covering the entire width of the pipe. The reason this was done was because for viewing angles that were greater than 60 degrees, there are effects related to non-observance of Lambert’s law. The bigger problem was the automatic start of the thermal imaging camera calibration, which caused abrupt changes in the measurements. This problem was solved by additionally measuring the area of the constant temperature outside the test object, but within the field of view of the camera. The measurements were recalculated from the change in temperature of the reference surface over time, which was constant.

## 4. Mathematical Model of the Temperature Distribution in a Single Tube Caused by Pulse Stimulation

The thermal wave equation is the foundation of contemporary active thermography [25]. In the case of a semi-finite body subjected to periodical thermal excitation, this equation makes it possible to determine the value of temperature at depth *z* [m], and at instant *τ* [s]:

(1) $$T\left(z,\tau \right)=T_{0}e^{-\frac{z}{\mu }}\;cos\left(\Omega \tau -\frac{2\pi z}{\lambda }\right)$$

where:

$T_{0}$—temperature on the sample surface (for *z* = 0) [K];
Ω—angular frequency of periodical thermal excitation [rad s^−1^];
*λ* = 2*πμ*—length of the thermal wave [m];
*µ* = 2αΩ—length of the diffusion path [m];
*α*—thermal diffusivity of the sample material [m^2^ s^−1^].

The most straightforward procedure to detect defects in the surface layer of material is pulsed thermography [15]. It requires knowledge of the time variation of excess temperature on the surface of the examined sample for thermal excitation, given as a Dirac impulse:

(2) $$\Delta T\left(\tau \right)=\frac{Q}{e\sqrt{\pi \tau }}$$

In Equation (2) the following notation is used:

Δ*T*—a rise of sample temperature [K];
*e* = $\sqrt{k\rho C_{p}}$—thermal effusivity of sample material [W m^−2^ K^−1^];
*k*—thermal conductivity [W m^−1^ K^−1^];
*ρ*—material density [kg m^−3^]
*C_p_*—specific heat of the material [J kg^−1^ K^−1^];
*Q*—density of impulse energy [m^−2^].

The existence of a defect in the surface layer of the material is indicated by the difference between the recorded temperature transient, and the transient obtained on the basis of relationship (2) for the measured value of material effusivity and the fixed value of the energy source.

The analytical solution to the previously described case may be found, e.g., in references [26,27]. The Fourier equation may be reduced to a dimensionless equivalent, when considering the geometry. Then, $T\left(z,\tau \right)$ corresponds to the temperature distribution at depth *z*, at the instant TAU, and is given by the following relationship:

(3) $$T\left(z,\tau \right)=T_{f}\left[1+\frac{2l}{\pi z_{e}}\overset{\infty }{\underset{n=1}{\sum }}\frac{1}{n}sin\frac{n\pi z_{e}}{l}cos\frac{n\pi z}{l}exp\left(\frac{-\tau \alpha^{2}\pi^{2}n^{2}}{l^{2}}\right)\right]$$

where:

$T_{f}=T_{0}\frac{z_{e}}{l}$—final temperature for the heat exchange process (for *τ*→∞).

Equation (3) follows that the analytical solution is rather complex, even for a simplified geometry. From the viewpoint of experimental implementation, acquiring either a periodic or an impulse excitation may be troublesome. Therefore, the step heating method was used in experimental tests within the present study.

## 5. Models and Results Analysis

### 5.1. Models Used for Calculations

**Figure 9:**
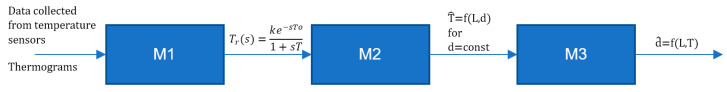
Steps of the analysis with marked primary input and output data.

The suggested method is similar to the one proposed in [28]. The locations of the pixels in the non-defective area are initially determined by an empirical rule based on a curve-fitting model; then, the thermal data of defective areas are selected for estimating defect depth. The difference is not in image comparison, but in the determination of time constants for pixels based on the step response. All results saved in the common database were analyzed by calculating three successive mathematical models, marked in Figure 9 as M1, M2, and M3. The results obtained from model M1 were used in the M2 and M3 models.

### 5.2. Model M1

In the M1 model, the transmittances of heat wave propagation in water and the pipe were approximated using delayed first-order inertia, described by the operator Equation (4), and then correlated with the location and wall thickness.

(4) $$T_{r}\left(s\right)=\frac{ke^{-sT_{o}}}{1+sT}$$

**Figure 10:**
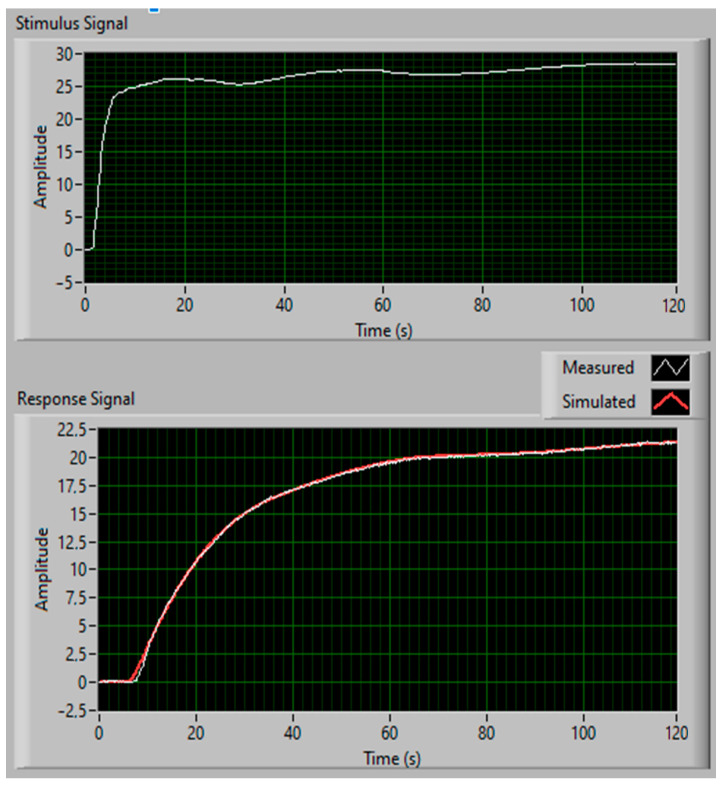
Thermal forcing, and real and approximated responses.

The transmittances were determined on the basis of the response to the quasi-step excitation. As a result, the transmittance parameters of $T_{r}$ were determined: gain, *k*, delay, $T_{o}$, and the time constant, *T*, at each analyzed measuring point. In all cases, the force was recorded by the TC1 thermocouple, and the responses were temperature changes recorded in selected places marked as TW11 … TW73, read from the recorded series of thermograms. For the calculation, we used the LabView software again. The Advanced Signal Processing Toolkit was used to estimate the parameters of the continuous transfer function model. The estimates were made based on the stimulus and response signal, using the SI Estimate Continuous Transfer Function Model. The quality of estimation with delayed first-order inertia is shown in Figure 10.

The real response and its approximation were almost the same. The root mean square error (RMSE) equaled 0.1269, when forced by a jump with a temperature of 28 °C.

**Figure 11:**
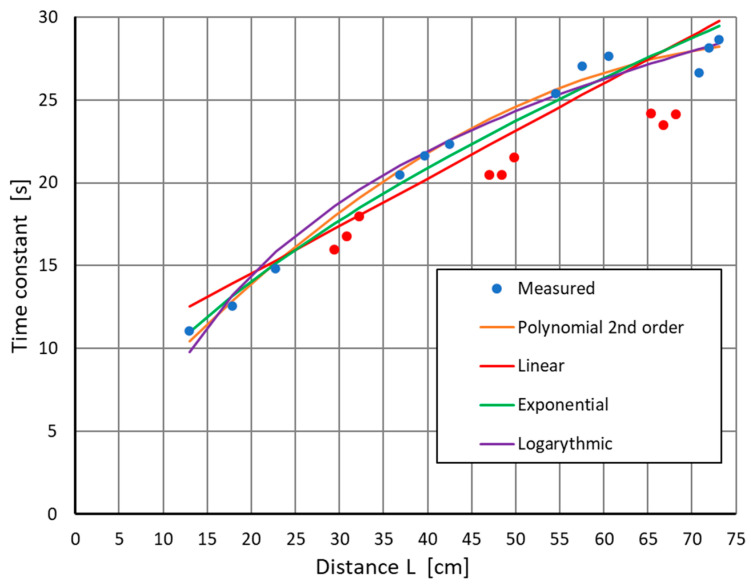
Changes in the time constant *T* depending on the distance from the input source *L* and the wall thickness of the tested object (blue points—*T* for *d* = 5 mm, red points—*T* for *d* = 3.5 mm, 4 mm, 4.5 mm).

In further analyses, the gain, *k*, and delay, *T_o_*, related to liquid transport in the test facility, were omitted; however, they could help correct the results. The focus was on time constant, *T*, depending on the object wall thickness and the distance from the point where TC1 was installed. Example results of the time constant *T* values, determined during the modelling of M1, are shown in Figure 11 (blue and red points).

As expected, the values of the time constant, *T*, depended on the distance, *L*, and the wall thickness of the test tube at a given point, *d*. Already at this point, it was noted that the time constants at points located on the narrows (red points) were located at a certain distance from the approximated line, which potentially allowed for locating places with a different thickness, *d*.

### 5.3. Model M2

Model M2 was based on approximating time-constant changes, depending on the location of the measurement point. In the first step, the model applies points for which the wall thickness is constant and equal to 5 mm. Four mathematical models were selected: linear, polynomial of the second order, logarithmic, and exponential, in order to choose the best one that reflected the observed changes. For one of the experiments, the obtained approximation plots are presented in Figure 11, in the form of lines.

The quality of the approximations obtained was assessed on the basis of two criteria: the coefficient of determination R^2^, and the root mean square error (RMSE). The results obtained for each experiment and the four models are summarized in Table 1.

The calculations showed that the best fit was obtained using a second-order polynomial model. The mean coefficient of determination R^2^ for this model was 0.981, and the RMSE was 0.624. Additionally, the significance of the correlation coefficient was checked by calculating the t-statistic, according to Equation (5), for each model.

(5) $$t=\frac{r\sqrt{n-2}}{\sqrt{1-r^{2}}}$$

A high probability level was adopted, *p* = 0.999, for which the critical value of $t_{n-2,\alpha }$ is 4.59. For all models, the calculated t-statistics were greater than the critical value, proving a statistically significant correlation. The lowest statistic for the least fitted linear model was *t* = 5.88. The second-order polynomial model was three times higher, and amounted to 17.96.

**Figure 12:**
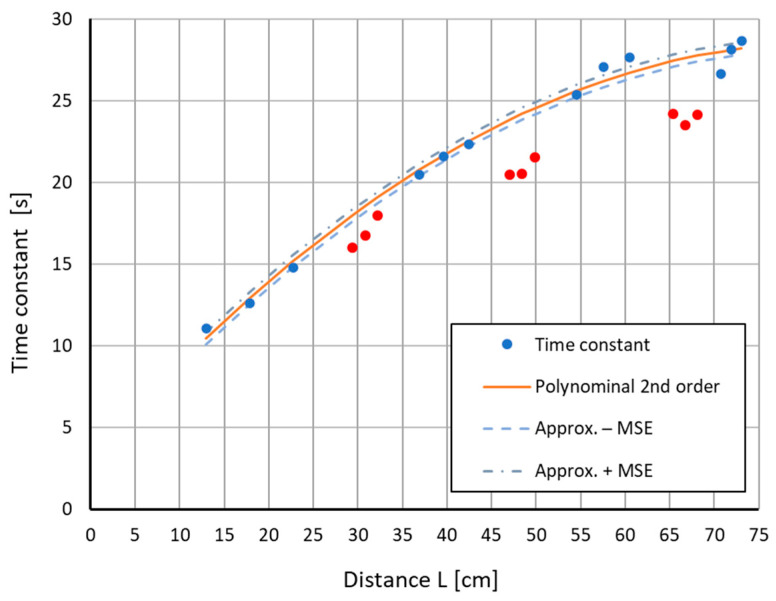
Approximation of the time constant $\hat{T}$, depending on the distance *L* for points with a wall thickness of 5 mm (blue points), and time constants for points with a smaller wall thickness (red points).

After determining the approximation parameters for each of the four tested models and for each experiment, the differences between the obtained results and the adopted model were calculated. Then, we checked which of the obtained results fell within the ±RMSE range around the approximating function. The graphical presentation of the results for one of the series of measurements is shown in Figure 12.

When the calculated difference exceeded ±RMSE, it was assumed that the pipe wall thickness decreased. Results of such identification (given in %) are shown in Table 2.

As can be seen, the model using second-order approximation identified 100% of the pipe wall thickness locations for each thickness reduction. The logarithmic model successfully detected changes in thickness from 1 mm upwards. The remaining models could detect changes from 1.5 mm. Unfortunately, for all of the models, there were erroneous detections of wall thickness reduction in places where it did not occur. Considering all measurement points (Table 2, columns “All”) for the second-order approximation, on average, one point in the series was identified incorrectly, resulting in a 7% incorrect identification rate. For others models, the obtained results were worse. However, this result is satisfactory, as is the case with screening tests. It is essential that no location with a thinner wall thickness be missed.

The M2 model allowed for an initial classification of the wall thickness of the tested pipe, based on the approximation of the change in the time constant $\hat{T}=f\left(L,d\right)$ that depended on the distance *L* and the pipe thickness *d*. For the second-order polynomial approximation, the model correctly identified places with a smaller pipe wall thickness. In a few cases, the algorithm indicated a point in the vicinity of the actual neck as a place with thinner walls.

### 5.4. Model M3

**Figure 13:**
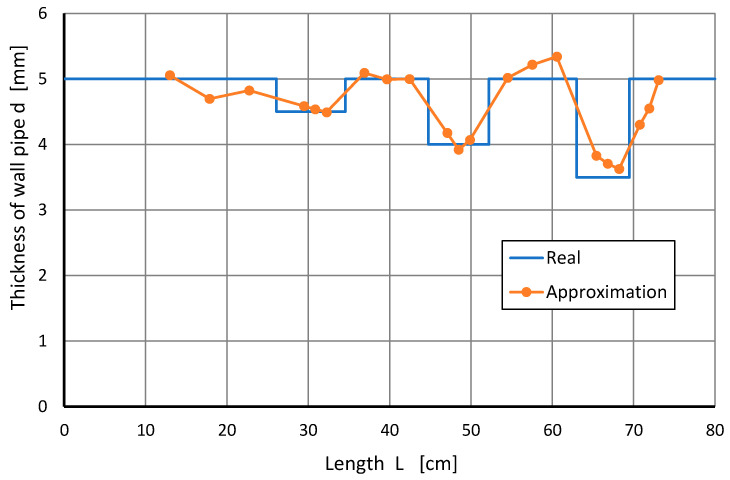
Fitting the measurement points for the applied second-order regression for experiment VIII.

Based on the same data, for the same types of models, the approximation $\hat{d}=f$(*L*,*T*) was calculated, allowing for the assessment of pipe wall thickness. An exemplary comparison of the actual wall thicknesses with those that were calculated on the basis of the model with a polynomial of the second-order is shown in Figure 13.

**Figure 14:**
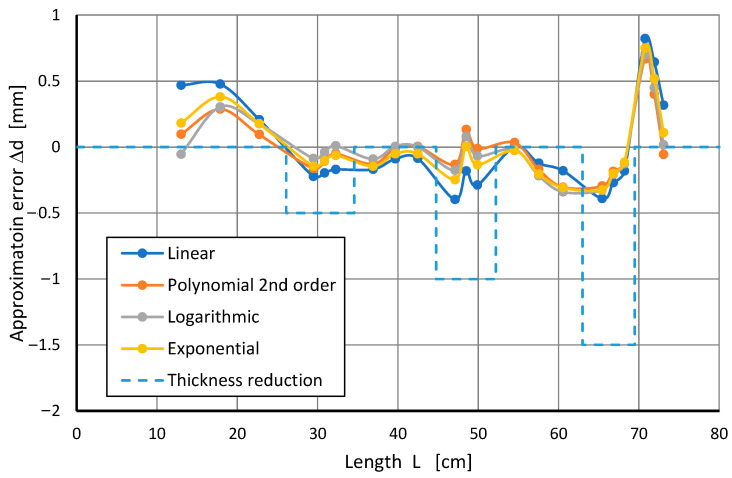
Approximation errors $\hat{d}$ for series VII of measurements.

Approximation errors (related to the actual pipe wall thickness) were calculated for a better visual assessment. The results of the exemplary series VII of measurements are shown graphically in Figure 14. It is noteworthy that for places of reduced thickness, the approximation errors usually have negative values. The diagram also shows the change in pipe wall thickness.

For a better assessment of the models used, approximation errors (related to the actual pipe wall thickness) were calculated, i.e., the maximum and minimum values of differences, as well as the RMSE. Outcomes for all measurements are presented in Table 3. Similarly to the M2 model, the best results were obtained for the second-order polynomial approximation model.

Additionally, the values of the approximation limit errors and RMSE were analyzed separately for the heating and cooling process of the tested object, the results of which are shown in Table 4. It is noteworthy that for the second-order model during heating, lower RMSE values were obtained. In this case, the RMSE value was 0.13 mm, and the maximum error did not exceed 0.54 mm, with the constriction being −0.32 mm. There were no significant differences for the other approximations.

Such results can be considered very promising, as they would allow the detection of even minor defects in wall thickness. A decision to replace a thermal screen is made when the thickness loss is 3 mm. Similarly to the M2 model, the second-order polynomial approximation led to the most satisfactory results.

## 6. Discussion of Results

This research was divided into three stages, with each of them being modelled as M1, M2, and M3. Within each of them, reference was made to four approximations of the obtained experimental data: linear, second-order polynomial, logarithmic, and exponential. In the first model, M1, the time constants at the given points were determined as a response to the applied excitation. These results already allowed for a visual assessment of the places where the wall thickness was less than the standard value of 5 mm, especially in places where the wall thickness was reduced by more than 1 mm. In some cases, the results were ambiguous for wall thicknesses that had reduced by 0.5 mm, as seen in Figure 11.

In model M2, it was quantified whether, for a given approximation, it is possible to identify places with reduced wall thickness. The M2 model, regardless of the approximation applied for each of the ten analyzed experiments, always detected a 1.5 mm change in wall thickness. The logarithmic approximation made it possible to detect a change in thickness of 1 mm, and the approximation using a second-order polynomial was able to detect even a 0.5 mm change in thickness. However, it should be noted that although the thickness reduction was always detected, there were also errors in detecting the standard wall thickness (5 mm), always occurring at the edge of a wall thickness variation. In the case of diagnostic tests, for which the developed method was dedicated, it is essential not to miss places with reduced thickness. Thus, since erroneous detection occurred at the edge of areas of varied thickness, the obtained result was considered satisfactory.

In the third model, attempts were made to determine the pipe wall thickness. Using M3 modelling, the estimated reduction in the pipe wall thickness for all models used, except for the linear one with a border error, did not exceed 0.5 mm. The best result was obtained for the approximation using a second-order polynomial during heating. The limit error was obtained at a level of 0.3 mm. All of the approximations used were within the error of thickness not exceeding 1 mm, and it should be remembered that a decision to replace a thermal screen is made when the thickness loss is 3 mm. However, accurate measurement is important because, based on the history of boiler testing in power plants, it is possible to forecast the dates of future remedies better.

The proposed method is dedicated to a specific facility where usually the research experiments can only be carried out once during a boiler renovation. Therefore, each measurement series was analyzed separately, and correct results were obtained for the best approximation (second-order polynomial).

## 7. Summary

Although the measurement of the wall thickness of metal pipes is not a technical problem, and many measurement methods are known, it is still a challenge to carry out for the heat shields of power boilers. First of all, currently known measurement methods are not made across their entire surface, but only at selected points. Secondly, full diagnostics require thousands of measurements, and there is no certainty that all of the areas that require repair will be detected. The conducted tests in this study are an introduction to the development of a method that allows for a preliminary diagnosis of the entire surface of a boiler screen. It will be able to be performed in parallel with the test of the patency of the screen pipes, which is also performed with the thermal vision method. The benefits of the proposed method include a reduction in the diagnosis time. If areas of a screen that requires replacement are detected, it is always possible to perform spot measurements, e.g., ultrasonic, in order to confirm them.

The developed method is so promising that an extension to the measurement station has been planned. For this purpose, a fragment of an actual boiler screen was obtained, in order to repeat the research using the authentic object. The data acquisition system also requires improvement, especially in automating the next steps in the proposed algorithms.

Although the proposed method has a dedicated application, it can be applied to any type of pipeline, as long as its surface is not thermally insulated. It is reasonable and purposeful to use artificial neural networks and deep learning, along [29] with data acquired in a described experiment, to classify furnace shield degradation, as using these methods may result in better outcomes.

## Figures and Tables

**Table 1:** Calculated coefficients of determination R^2^ and RMSE of model fit.

Series	Mode	Input Step	Coefficient of Determination R^2^				RMSE for Model			
			Linear	2nd Order	Logarithmic	Exponential	Linear	2nd Order	Logarithmic	Exponential
III	HEATING	45.0	0.776	0.976	0.905	0.853	1.949	0.638	1.272	1.580
V	HEATING	46.6	0.802	0.978	0.920	0.859	2.148	0.710	1.361	1.813
VI	COOLING	21.3	0.944	0.990	0.980	0.972	1.424	0.608	0.851	1.014
VII	HEATING	46.5	0.778	0.987	0.912	0.867	1.612	0.390	1.015	1.249
VIII	COOLING	31.6	0.883	0.970	0.953	0.936	1.388	0.702	0.880	1.024
IX	COOLING	17.2	0.920	0.981	0.971	0.956	1.561	0.765	0.949	1.160
X	HEATING	47.5	0.777	0.982	0.909	0.854	1.890	0.541	1.205	1.525
XI	COOLING	32.0	0.912	0.986	0.978	0.955	1.472	0.579	0.741	1.056
XII	COOLING	17.3	0.919	0.982	0.971	0.954	1.479	0.701	0.884	1.114
XIII	COOLING	34.5	0.826	0.979	0.940	0.897	1.745	0.601	1.028	1.343
Avg			0.854	0.981	0.944	0.910	1.667	0.624	1.019	1.288

On the green-yellow-red gradient scale for R^2^, the green background color indicates the maximum desirable values and the red one indicates the minimum values. On the blue-red-blue gradient scale for RMSE, the blue background color indicates the minimum desirable values, and the red indicates the maximum values.

**Table 2:** Calculated indexes of identifying points in places with reduced wall thickness.

Series	Linear				Polynomial 2nd Order				Logarithmic				Exponential			
	Decrease in Thickness				Decrease in Thickness				Decrease in Thickness				Decrease in Thickness			
	All	0.5 mm	1 mm	1.5 mm	All	0.5 mm	1 mm	1.5 mm	All	0.5 mm	1 mm	1.5 mm	All	0.5 mm	1 mm	1.5 mm
	%															
III	57.1	0	0	100	95.2	100	100	100	85.7	66.7	100	100	71.4	0	66.7	100
V	57.1	0	0	100	95.2	100	100	100	85.7	66.7	100	100	66.7	0	66.7	100
VI	76.2	0	100	100	95.2	100	100	100	90.5	100	100	100	85.7	66.7	100	100
VII	57.1	0	0	100	95.2	100	100	100	81.0	66.7	100	100	71.4	0	100	100
VIII	66.7	0	100	100	95.2	100	100	100	90.5	100	100	100	81.0	33.3	100	100
IX	71.4	0	66.7	100	90.5	100	100	100	95.2	100	100	100	81.0	33.3	100	100
X	71.4	0	100	100	90.5	100	100	100	81.0	33.3	100	100	76.2	0	100	100
XI	71.4	0	100	100	90.5	100	100	100	81.0	33.3	100	100	76.2	0	100	100
XII	71.4	0	100	100	90.5	100	100	100	81.0	33.3	100	100	76.2	0	100	100
XIII	71.4	0	100	100	90.5	100	100	100	81.0	33.3	100	100	76.2	0	100	100
AVG	67.1	0	66.7	100	92.9	100	100	100	85.2	63.3	100	100	76.2	13.3	93.3	100

On the green-yellow-red gradient scale, the green background color indicates the maximum desirable values and the red one indicates the minimum values.

**Table 3:** Limit errors of approximation $\hat{d}$ and RMSE for the four approximation methods.

Series	Linear			Polynomial 2nd Order			Logarithmic			Exponential		
	Dd_max_	Dd_min_	RMSE	Dd_max_	Dd_min_	RMSE	Dd_max_	Dd_min_	RMSE	Dd_max_	Dd_min_	RMSE
	mm											
III	0.85	−0.57	0.42	0.53	−0.28	0.17	0.80	−0.40	0.31	0.83	−0.48	0.36
V	0.86	−0.55	0.42	0.54	−0.28	0.19	0.80	−0.35	0.31	0.85	−0.46	0.36
VI	0.83	−0.46	0.36	0.53	−0.38	0.20	0.59	−0.50	0.25	0.75	−0.38	0.27
VII	0.79	−0.56	0.42	0.39	−0.32	0.15	0.71	−0.39	0.30	0.75	−0.45	0.35
VIII	0.82	−0.40	0.34	0.67	−0.30	0.22	0.70	−0.34	0.24	0.75	−0.33	0.27
IX	0.88	−0.56	0.39	0.61	−0.52	0.26	0.70	−0.54	0.29	0.82	−0.50	0.32
X	0.77	−0.57	0.40	0.38	−0.23	0.14	0.67	−0.41	0.29	0.72	−0.48	0.34
XI	0.83	−0.61	0.38	0.53	−0.38	0.21	0.66	−0.39	0.23	0.77	−0.49	0.29
XII	0.69	−0.50	0.35	0.38	−0.49	0.23	0.47	−0.51	0.25	0.59	−0.46	0.28
XIII	0.81	−0.51	0.39	0.48	−0.39	0.20	0.71	−0.39	0.26	0.76	−0.42	0.31
AVG	0.81	−0.53	0.38	0.50	−0.36	0.20	0.68	−0.42	0.27	0.76	−0.45	0.31
Max mod.	0.88	−0.61	0.42	0.67	−0.52	0.26	0.80	−0.54	0.31	0.85	−0.50	0.36

On the red-yellow-green gradient scale, the green background color indicates the minimum desirable values and the red one indicates the maximum values.

**Table 4:** Limiting errors of approximation, $\hat{d}$ and RMSE, for the estimation of the tube wall thickness for different types of excitations, using different M3 models.

Step Force	Linear			Polynomial 2nd Order			Logarithmic			Exponential		
	Dd_max_	Dd_min_	RMSE	Dd_max_	Dd_min_	RMSE	Dd_max_	Dd_min_	RMSE	Dd_max_	D_dmin_	RMSE
	mm											
HEATING	0.86	−0.57	0.33	0.54	−0.32	0.13	0.80	−0.41	0.24	0.85	−0.48	0.28
COOLING	0.88	−0.61	0.37	0.67	−0.52	0.22	0.71	−0.54	0.25	0.82	−0.50	0.29

On the red-yellow-green gradient scale, the green background color indicates the minimum desirable values and the red one indicates the maximum values.

## Data Availability

The data presented in this study are available on request from the corresponding author. The data are not publicly available, due to large amounts of data being in the form of video files and tables.

## References

[1] Dzierżanowski Ł., Tomaszewski M. Matching the metrological databases of deterioration areas with the diagnostic data based on the OP-650 power boiler shields example. Proceedings of the XIII Conference Computer Applications in Electrical Engineering.

[2] Zator S., Krawczyk M. (2009). Integration of diagnostic databases with spatial model. Pozn. Univ. Technol. Acad. J..

[3] Hardy T., Arora A., Pawlak-Kruczek H., Rafajłowicz W., Wietrzych J., Niedźwiecki Ł., Vishwajeet, Mościcki K. (2021). Non-Destructive Diagnostic Methods for Fire-Side Corrosion Risk Assessment of Industrial Scale Boilers, Burning Low Quality Solid Biofuels—A Mini Review. Energies.

[4] Ma Q., Tian G., Zeng Y., Li R., Song H., Wang Z., Gao B., Zeng K. (2021). Pipeline In-Line Inspection Method, Instrumentation and Data Management. Sensors.

[5] Vakhguelt A., Kapayeva S.D., Bergander M.J. (2017). Combination Non-Destructive Test (NDT) Method for Early Damage Detection and Condition Assessment of Boiler Tubes. Procedia Eng..

[6] Kumar D., Mishra S. (2015). High Temperature Ultrasonics Testing for In-situ Condition Monitoring of Superheated Steam. Int. J. Sci. Res..

[7] Boynard C., Lopes E.D., Estanislau S., Pimenta G. Influence of Superficial Scale in Signal Variation Generated by EMAT on Boiler Tube Inspection. Proceedings of the 9th European Conference on NDT.

[8] Bergander M.J. (2003). EMAT thickness measurement for tubes in coal-fired boilers. Appl. Energy.

[9] Ho M., El-Borgi S., Patil D., Song G. (2020). Inspection and monitoring systems subsea pipelines: A review paper. Struct. Health Monit..

[10] Innospection SLOFEC TM—Fast Corrosion Screening Technique. http://www.ndttechnologies.com/Brochures/Tank%20Scanning/SLOFEC%20Technique.pdf.

[11] Cheng W. (2012). Pulsed eddy current testing of carbon steel pipes wall-thinning through insulation and cladding. J. Nondestruct. Eval..

[12] Boateng A., Danso K.A., Dagadu C.P.K. (2013). Non-Destructive Evaluation Of Corrosion On Insulated Pipe Using Tangential Radiographic Technique. Int. J. Sci. Technol. Res..

[13] Mayet A.M., Chen T.-C., Ahmad I., Tag Eldin E., Al-Qahtani A.A., Narozhnyy I.M., Guerrero J.W.G., Alhashim H.H. (2022). Application of Neural Network and Dual-Energy Radiation-Based Detection Techniques to Measure Scale Layer Thickness in Oil Pipelines Containing a Stratified Regime of Three-Phase Flow. Mathematics.

[14] Bagavathiappan S., Lahiri B.B., Saravanan T., Philip J., Jayakumar T. (2013). Infrared thermography for condition monitoring—A review. Infrared Phys. Technol..

[15] Garrido I., Lagüela S., Otero R., Arias P. (2020). Thermographic methodologies used in infrastructure inspection: A review—Post-processing procedures. Appl. Energy.

[16] Tian G.Y., Gao Y. (2016). Multiphysics Integrated NDT&E and Progress on Eddy Current Puled Thermography, Electromagnetic Nondestructive Evaluation (XIX).

[17] Oliveira B.C.F., Seibert A.A., Borges V.K., Albertazzi A., Schmitt R.H. (2021). Employing a U-net convolutional neural network for segmenting impact damages in optical lock-in thermography images of CFRP plates. Nondestruct. Test. Eval..

[18] Wang Z., Zhu J., Tian G., Ciampa F. (2019). Comparative analysis of eddy current pulsed thermography and long pulse thermography for damage detection in metals and composites. NDT E Int..

[19] Huang Z., Zhu J., Zhuo L., Li C., Liu C., Hao W., Xie W. (2022). Non-destructive evaluation of uneven coating thickness based on active long pulse thermography. NDT E Int..

[20] Zhuo L., Yang X., Zhu J., Huang Z., Chao J., Xie W. (2023). Size determination of interior defects by reconstruction of subsurface virtual heat flux for step heating thermography. NDT E Int..

[21] EPRI Line Scanning Thermography for Evaluation of Boiler Tube Overlay Disbond, Product ID 1020523, 21 December 21 2009, Technical Update, EPRI Project Manager Zayicek P. https://www.epri.com/research/products/000000000001020523.

[22] Dudzik S. (2013). Characterization of material defects using active thermography and an artificial neural network. Metrol. Meas. Syst..

[23] Łopata S., Kocot M. (2017). The conditions for thermographic testing of thermal power engineering installations. Tech. Trans..

[24] Zator S., Lasar M. (2012). Reconstruction of high-resolution thermal images on the basis of standard thermal images. Pomiary Autom. Kontrola.

[25] Dudzik S. (2010). Approximation of thermal background applied to defect detection using the methods of active thermography. Metrol. Meas. Syst..

[26] Maldague X.P. (2001). Theory and Practice of Infrared Technology for Non-Destructive Testing.

[27] Carlslaw H.S., Jaeger J.C. (1959). Conduction of Heat in Solids.

[28] Zhang Y., Zhang K., Wang W., Shu S., Zhang Y., Lang X., Chen J. (2022). Effect of Background Subtraction on Defect Detection in Thermographic Signal Reconstruction Coefficient Images. J. Nondestruct. Eval..

[29] Michalski P., Ruszczak B., Tomaszewski M. Convolutional Neural Networks Implementations for Computer Vision: Biomedical Engineering and Neuroscience. Proceedings of the 3rd International Scientific Conference on Brain-Computer Interfaces.

